# Growth, Feed Utilization, Lipid Metabolism, and Metamorphosis of Bullfrog (*Aquarana catesbeianus*) Tadpoles Fed Diets With Different Lipid Levels

**DOI:** 10.1155/2024/5513496

**Published:** 2024-11-02

**Authors:** Juan Gao, Ling Wang, Jian Zhang, Kangle Lu, Kai Song, Xueshan Li, Chunxiao Zhang

**Affiliations:** Xiamen Key Laboratory for Feed Quality Testing and Safety Evaluation, Fisheries College, Jimei University, Xiamen 361021, China

## Abstract

This study investigated the effect of dietary lipid levels on growth performance, lipid metabolism, antioxidant capacity, digestive enzyme activity, and metamorphosis rate of bullfrog (*Aquarana catesbeianus*) tadpoles. A total of six isonitrogenous diets were prepared, each containing 4.46% (L5), 6.95% (L7), 9.10% (L9), 10.90% (L11), 12.34% (L13), and 15.00% (L15) crude lipid content. The experimental diets were administered to triplicates of tadpoles (stage 25, 0.007 g) twice daily for 75 days with a daily feeding rate of 6.50% of their body weight. Weight gain (WG), specific growth rate (SGR), crude protein content of the whole body, apparent digestibility of dry matter and gross energy, intestinal lipase (LPS) capacity, lipoprotein lipase (LPL), and carnitine palmitoyltransferase-I (CPT-I) and contents of growth hormone (GH) and insulin-like growth factor-1 (IGF-1) in the liver, high-density lipoprotein cholesterol (HDL-C) content in the serum, and metamorphosis rate at stages 40 and 41 increased as the dietary lipid level increased from 4.46% to 12.34% and then decreased. As the dietary lipid level increased from 4.46% to 10.90%, the protein efficiency ratio (PER), protein deposition rate (PDR), lipid deposition rate (LDR), crude lipid content and gross energy of the whole body, apparent digestibility of the crude lipid, superoxide dismutase (SOD), catalase (CAT) activities in the liver, and the intestinal trypsin (TPS) activity all increased and then decreased. According to the second-order polynomial regression analysis of the WG and metamorphosis rate at stage 41 to the dietary lipid level, the ideal dietary lipid content for tadpoles was 11.08% and 10.72%, respectively. Overall, the appropriate dietary lipid level for bullfrog tadpoles was found to be 10.72%–11.08% of the diet.

## 1. Introduction

Lipid is a vital energy source in feed and is responsible for the development and growth of animals by supplying essential fatty acids (EFAs), phospholipids, and sterols [1, 2]. Deficiency of dietary lipids will result in slowed growth, weakened disease resistance, and direct effects on immunity [3, 4]. The immunity of the body is compromised by excessive lipid deposition in the liver, which endangers health, while excessive levels of dietary lipids result in a metabolically burdensome condition [5, 6]. Thus, it is crucial to determine an optimal dietary lipid level to enhance protein consumption, facilitate energy provision, and optimize the growth of aquatic animals.

The bullfrog (*Aquarana catesbeianus*) is recognized as one of the most desirable amphibians and holds significant value in the global marketplace due to its commercial and economic importance [7, 8]. During the different stages of bullfrog growth, the rearing of bullfrog tadpoles plays a crucial role in the development of the frog larvae. Studying the nutrition of bullfrog tadpoles is of utmost importance due to their dependence on inadequate commercial feeds in traditional tadpole cultures for other aquatic animals [9, 10]. Therefore, it is necessary to quantify the nutritional requirements of the tadpoles of bullfrogs. Several recent studies have focused on the protein requirements of bullfrog tadpoles [9, 11, 12]. Our previous study has determined that the ideal protein levels for bullfrog tadpoles were 42.49%–46.50% of their diet [13]. Surprisingly, no study to date offers comprehensive data regarding the nutritional requirements of bullfrog tadpoles, specifically their lipid requirements.

It is necessary to investigate the nutritional requirements of bullfrog tadpoles on synthetic diets. Specifically, the optimal lipid levels are of significant importance to the growth, health, metamorphosis, and physiological metabolism of bullfrog tadpoles. Therefore, this study aimed to determine the impact of dietary lipid levels on lipid metabolism, digestive enzyme activities, and metamorphosis rate. The result of this study will offer useful information for the development of artificial diets for bullfrog tadpoles.

## 2. Materials and Methods

### 2.1. Ethics Statement

This research followed guidelines of the Animal Care Advisory Committee of Jimei University (Approval No. 2019-0906-003).

### 2.2. Experimental Diets

Approximately six isonitrogenous (45.50%) diets were prepared which comprised fish meal, soy protein concentrates as protein sources, soybean oil as lipid sources, and wheat flour as the carbohydrate sources, as illustrated in Table 1. The lipid levels identified as 4.46% (L5), 6.95% (L7), 9.10% (L9), 10.90% (L11), 12.34% (L13), and 15.00% (L15), respectively. All ingredients were processed using grinding and sieving via a 250 µ screen. Dry matter was thoroughly mixed with all feed ingredients. Water was added, and toppings were recombined. Afterward, the TSE-65 twin-screw extruder was used to produce 1.0 mm expanded pellets. The granules were evenly coated with oil, dried at 45°C for 8 h, grounded, and stored at −20°C until further analysis.

### 2.3. Experimental Bullfrog Tadpoles and Feeding Trial

In this study, bullfrog tadpoles were obtained from a merchant breeding facility (Xiamen, Fujian province, China) and were derived from an identical tadpole colony. The feeding trial was conducted in an aquaculture system at Jimei University. At the end of the acclimatization phase, 900 healthy stage 25 bullfrog tadpoles [15] with a mean weight of 0.007 g were dumped into 18 glass aquaria (50 cm × 45 cm × 30 cm) that were filled with 50 L of water. Each aquarium was densified with 50 tadpoles. In the feeding experiments, the tadpoles were fed twice daily at 8:30 and 17:30, at a rate of 6.5% of their body weight, for 75 consecutive days. Each treatment was replicated thrice. To regulate the quantity of nutrition, tadpoles in each aquarium have been weighed weekly. The feed was supposed to be ingested within 1 h, and the feces and the remaining feed were removed using a siphon hose. Afterward, 70% of the water was exchanged. Feed intake (FI) was calculated via daily recording. During the development period, the following parameters were maintained: water temperature, 25−26°C; pH, 7.0–8.0; 12 h (light:dark) photoperiod; and dissolved oxygen, 5.5–7.0 mg/L.

## 3. Experimental Analysis

### 3.1. Sample Collection

The growth phase was recorded in the 4th week of the feed experiment. Before collecting samples, bullfrog tadpoles were starved of food for 24 h. The tadpole tissues were harvested using MS-222 (1:10,000, Sigma, St. Louis, MO, USA). The survivability, weight gain (WG), and metamorphosis rate of all bullfrog tadpoles in each aquarium were identified by counting and weighing each tadpole. Six bullfrog tadpoles were collected from each aquarium and stored at −20°C to analyze their entire body composition. All of the following sample collection procedures were performed on ice. Blood was collected from the tails of 20 bullfrog tadpoles in each aquarium using 75-μL capillary micropipettes, transferred to aseptic tubes, and stored at 4°C overnight. For biochemical indexes, the serum was separated after centrifugation at 3000 rpm for 10 min at 4°C and stored at −80°C. The livers of 20 bullfrogs from each aquarium were harvested, weighed, and stored at −80°C for further biochemical parameter analysis. The belly fat of these bullfrog tadpoles was disseminated and weighed in each aquarium to calculate the abdominal fat percentage (AFP). Intestine samples were collected for digestive enzyme activities analysis. The intestine was homogenized according to the procedures outlined in the previous study [16].

Feces were collected after 3 h of daily feeding and water changes and were siphoned into 300-mesh filter bags and quickly frozen at −20°C until analyzed. Feces were collected for 40 days before sampling. The calculation of apparent digestibility coefficients (ADCs) of dry matter, crude protein, crude lipid, and gross energy of the experimental feeds was described as per the previous method [17].

### 3.2. Chemical Composition

The feed, whole body, and feces were analyzed for their crude lipid, moisture, and ash contents using established standard methods [18]. The moisture content was determined by the oven-drying process until a stable weight was achieved at 105°C. Crude protein was measured using the Dumas combustion method (FP828P, LECO, USA). Crude fat was extracted using ether, while the ash was obtained via burning at 550°C for 8 h. Lastly, the gross energy was measured using a fully automated oxygen bomb calorimeter (Parr 6300; Parr Instrument Co., Moline, IL, USA). The yttrium content of the samples was quantitatively determined by inductively coupled plasma atomic emission spectroscopy (ICP-OES) (Leeman, USA).

### 3.3. Liver Histology

Liver tissues were dehydrated in a sugar solution and embedded in an optimal cutting temperature (OCT) encapsulation medium. They were then stained with Oil Red O as per the protocol of the previous study [19]. These tissues were sectioned into 10 µm using a cryosectioning machine (CryoStar NX50, Thermo Scientific, USA). These tissues were stained with Oil Red O and hematoxylin and visualized under a laboratory light microscope (Leica DM5500B, Solms, Germany).

### 3.4. Biochemistry Analyses

The levels of total cholesterol (T-CHO), triglyceride (TG), high-density lipoprotein cholesterol (HDL-C), and low-density lipoprotein cholesterol (LDL-C) in the serum were measured using biochemical assay kits (Jiancheng Biotech. Co., Nanjing, China), based on the provided instructions.

Liver samples were homogenized using 10 volumes (w/v) of ice-cold 0.86% sodium chloride (NaCl). Afterward, samples were centrifuged for 10 min at 2000 × *g* for further analysis. Protein concentration was quantified via the bicinchoninic acid (BCA) protein quantification reagent (Solarbio Co., Beijing, China). The antioxidant parameters (the levels of total antioxidant [T-AOC], malondialdehyde [MDA], superoxide dismutase [SOD], and catalase [CAT]) and hepatic enzymes related to lipoprotein lipase (LPL) in the liver were determined by using biochemical assay kits (Jiancheng Biotech. Co., Nanjing, China).

The activities of lipase (LPS), amylase (AMS), and trypsin (TPS) were visualized and quantified as per the manufacturer's instructions using biochemical assay kits (Jiancheng Biotech. Co., Nanjing, China). One unit of LPS activity is equivalent to the volume of enzyme required to hydrolyze 1 µmol of substrate per minute at 37°C. The quantity of protein that hydrolyzes 10 mg of starch/half hour at 37°C is referred to as one unit of AMS activity. One unit of enzyme activity is equivalent to 0.003 units of TPS/ng of protein when measured as a change in absorbance/min.

The levels of acetyl-CoA carboxylase (ACC), carnitine palmitoyltransferase-I (CPT-I), insulin-like growth factor-I (IGF-I), and growth hormone (GH) in the liver and thyroxine (T4) in the serum were determined by a competitive method using amphibian enzyme-linked immunosorbent kits (Jiancheng Biotech. Co., Nanjing, China).

### 3.5. Statistical Analysis

All diets were administered in a strictly controlled randomized design. The Shapiro–Wilk and Kolmogorov–Smirnov variance tests demonstrated that all values were consistent with heteroscedasticity and normalism. All data were statistically analyzed using Tukey's multiple range test and one-way analysis of variance (ANOVA), followed by the evaluation of the significance of linear or quadratic models via orthogonal polynomial comparisons. A statistically significant difference was indicated when *p* ≤ 0.05. All data were presented as mean ± standard errors of the means (SEM) via SPSS 25.0 software (Chicago, IL, USA).

## 4. Results

### 4.1. Growth Performance and Feed Utilization

**Figure 1:**
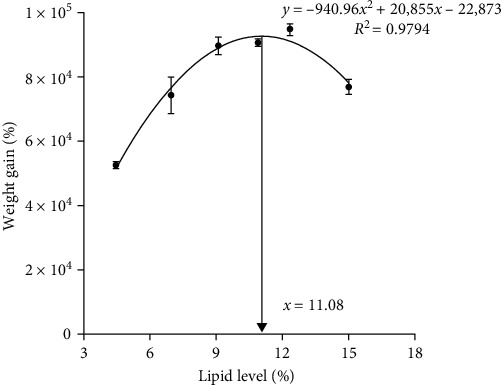
Second-order regression analysis between weight gain and dietary lipid levels for bullfrog tadpoles. Data were measured as mean ± standard errors of the means (SEM) (*n* = 3 for treatment).

As dietary lipid levels increased from 4.46% to 12.34%, WG and specific growth rate (SGR) increased and then decreased (Table 2). Feed conversion ratio (FCR) and FI of bullfrog tadpoles decreased and then increased with increasing levels of dietary lipids, and those of bullfrog tadpoles in group L9 were the lowest. Protein efficiency ratio (PER), protein deposition rate (PDR), and lipid deposition rate (LDR) increased as dietary lipid levels increased from 4.46% to 10.90% and then decreased. In addition, a slight decrease was observed in the survival of bullfrog tadpoles due to the increased lipid levels in the experimental diets. Using a second-order polynomial regression curve *y* = −940.96*x*^2^ + 20,855*x* − 22,873 (*R*^2^ = 0.9794), the optimal dietary lipid level based on WG can be determined to be 11.08% (Figure 1).

### 4.2. Body Composition and Morphological Parameters

The moisture of bullfrog tadpoles decreased as the dietary lipid level increased from 4.46% to 10.90% and then increased (Table 3). The crude protein of bullfrog tadpoles increased as the dietary lipid level increased from 4.46% to 12.34% and then decreased. The crude lipid, ash content, and gross energy increased as the dietary lipid level increased from 4.46% to 10.90% and then decreased.

In relation to body index, the hepatosomatic index (HSI) and AFP of tadpoles showed an initial increase followed by a decrease as dietary lipid levels increased. Tadpoles fed diets with L11 and L13 had the highest values for HSI and AFP, respectively. Further, the condition factor (CF) of bullfrog tadpoles increased gradually with the increase of dietary lipid levels from 4.46% to 15.00%.

### 4.3. Digestive Enzyme Activity and Nutrient Apparent Digestibility

The intestinal TPS and LPS activities increased initially and then decreased as dietary lipid levels upregulated. The highest values of TPS and LPS activities were observed in tadpoles that were fed diets with L11 and L13, respectively. Tadpoles that were fed diets with L13–L15 showed a higher intestinal AMS activity than those that were fed diets with L5–L11 (Table 4).

The ADCs for dry matter, crude lipid, and gross energy in bullfrog tadpoles showed a pattern of initially increasing and then decreasing with higher dietary lipid levels. The maximum ADCs were observed for L13, L11, and L13, respectively. However, there was a slight decrease in ADCs of proteins as the dietary lipid levels increased from 4.46% to 15.00%.

### 4.4. Serum Biochemical Indicators

**Figure 2:**
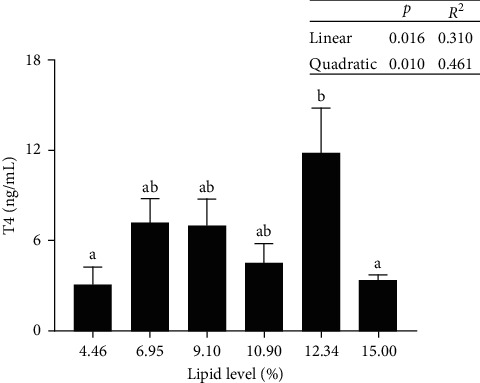
Amphibia thyroxine (T4) content in the serum of bullfrog tadpoles. Data were measured as mean ± standard errors of the means (SEM) (*n* = 3 for treatment). The values with different superscript letters indicate significant differences at *p* < 0.05.

The serum T-CHO, TG, and LDL-C contents of tadpoles had no significant differences among all interventions. HDL-C levels in serum increased as dietary lipid levels increased (Table 5). Further, serum T4 levels increased as dietary lipid levels increased from 4.46% to 12.34% and then decreased (Figure 2).

### 4.5. Liver Biochemical Indicators and Histological Examination

**Figure 3:**
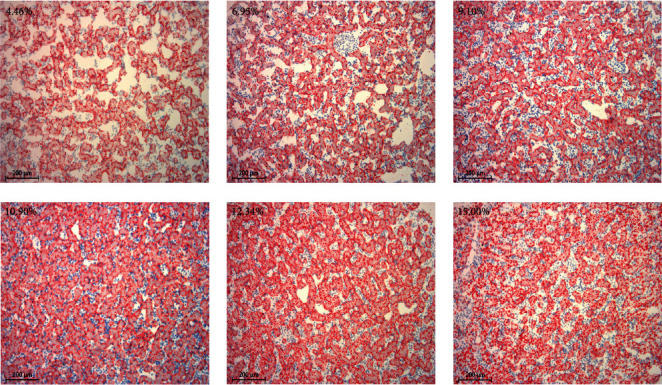
Histological examination of the liver in bullfrog tadpoles via Oil Red O staining.

The ACC activity in the liver of bullfrog tadpoles showed a decreasing trend with increasing dietary lipid levels; however, the activities of LPL and CPT-I showed an upward trend (Table 6). Furthermore, Oil Red O staining showed more lipid accumulation phenomenon in tadpoles fed diets with L11–L15 compared to those fed diets with L5–L9 (Figure 3).

**Figure 4:**
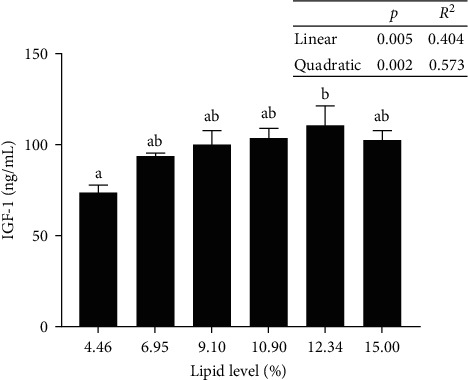
Insulin-like growth factor-1 (IGF-1) content in the liver of bullfrog tadpoles. Data were measured as mean ± standard errors of the means (SEM) (*n* = 3 for treatment). The values with different superscript letters indicate significant differences at *p* < 0.05.

**Figure 5:**
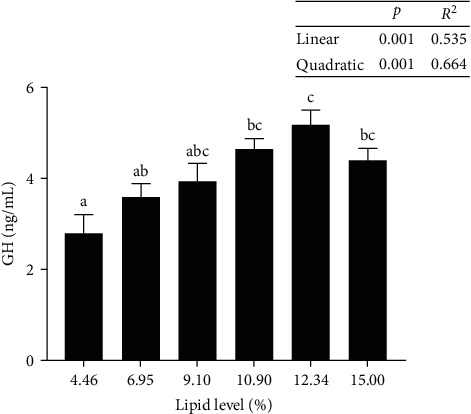
Growth hormone (GH) content in the liver of bullfrog tadpoles. Data were measured as mean ± standard errors of the means (SEM) (*n* = 3 for treatment). The values with different superscript letters indicate significant differences at *p* < 0.05.

In response to increasing dietary lipid levels, the level of T-AOC and the activities of SOD and CAT initially increased and eventually reduced. The maximum values were observed in tadpoles that were fed diets with L7, L11, and L11, respectively. The MDA levels did not show any substantial difference among all treatments (Table 6). IGF-I levels in the livers of tadpoles fed diet with L13 were significantly higher than those of tadpoles fed diet with L5 (Figure 4). Tadpoles fed diet with L13 showed substantially higher GH levels in their livers than those fed diets with L5 and L7 (Figure 5).

### 4.6. Metamorphosis Rate at Stage 40 and Stage 41

**Figure 6:**
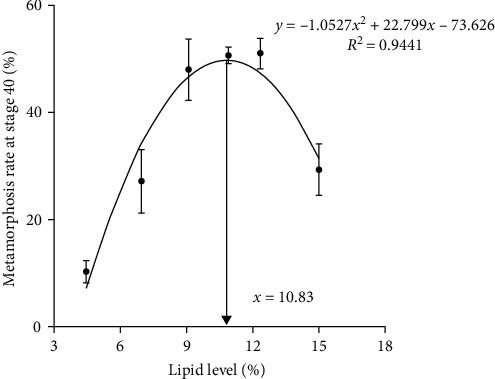
Second-order regression analysis between metamorphosis rate at stage 40 (including stage 41) and dietary lipid levels for bullfrog tadpoles. Data were measured as mean ± standard errors of the means (SEM) (*n* = 3 for treatment).

**Figure 7:**
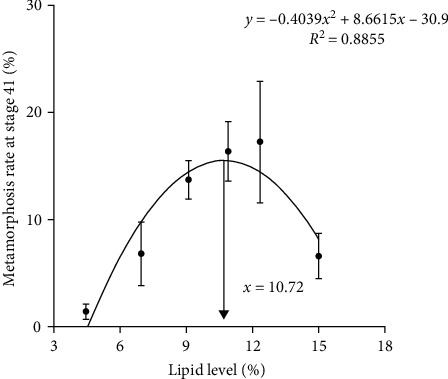
Second-order regression analysis between metamorphosis rate at stage 41 and dietary lipid levels for bullfrog tadpoles. Data were measured as mean ± standard errors of the means (SEM) (*n* = 3 for treatment).

The rate of metamorphosis at stages 40 and 41 initially increased and then decreased as dietary lipid levels increased. The maximal value of both was observed in tadpoles that were fed diet with L13. The metamorphosis rate was most favorable at growth stage 40, with a dietary lipid level of 10.83% (*y* = −1.0527*x*^2^ + 22.799*x* − 73.626, *R*^2^ = 0.9441) (Figure 6) and at growth stage 41, with a dietary lipid level of 10.72% (*y* = −0.4039*x*^2^ + 8.6615*x* − 30.9, *R*^2^ = 0.8855) (Figure 7). These data were obtained using second-order polynomial regression analysis.

## 5. Discussion

The current study demonstrated that bullfrog tadpoles' growth rate was enhanced by the consumption of a diet containing 9.10% to 12.34% lipids. This implied that their growth and development were dependent on the presence of an appropriate dietary lipid level. The metabolic organs may have been burdened by the excess lipids, which inhibited the growth of the tadpoles, resulting in a reduction in WG and SGR in tadpoles for lipid levels exceeding 12.34%. Generally, appropriate dietary lipid levels could maximize the use of protein for the growth of aquatic animals [20]. Moreover, bullfrog tadpoles fed 10.90% lipid had higher PER and PDR, suggesting that appropriate dietary lipid levels promote the absorption and consumption of feed nutrients in bullfrog tadpoles. Similar findings were also found in *Lateolabrax japonicus* [20], *Solea solea* juvenile [4], and *Ctenopharyngodon idella* juvenile [5]. Moreover, the initial size and weight of the froglets are considered to be critical factors in the efficacy of rearing [21]. Therefore, the rate of tadpole fattening and growth was influenced by premetamorphic WG. The rate of WG, as determined by second-order polynomial regression analysis, indicated that the optimal dietary lipid level for bullfrog tadpoles was 11.08%.

GH is a multipotent hormone secreted by the vertebrate pituitary gland [22]. It is implicated in major physiological processes in the body, including lipid and protein metabolism and bone and soft tissue growth. Furthermore, in most vertebrates, IGF-I is the primary growth agent of the GH/IGF axis [22]. The growth of somatic cells was shown to be regulated by the IGF system in response to variations in nutritional status [23]. The results indicated that bullfrog tadpoles that were fed a diet with a lipid level of 12.34% showed the highest levels of IGF-I and GH in their livers as well as better growth performance. It indicated that the growth rate of bullfrog tadpoles was closely associated with the changes in IGF-I and GH contents.

Fat consumption is restricted in aquatic animals, and fat accumulation in the tissues may result from an excessive amount of fat in the diet [24]. The results revealed that the morphology of tadpoles was slightly affected by the dietary lipid levels. The whole-body crude protein and lipid contents of bullfrog tadpoles increased, while the dietary lipid level increased from 4.46% to 10.90% and then decreased. This suggested that the crude protein and lipid contents were affected by different lipid levels in diets, and similar results were also found in *Seriola dorsalis* juvenile [25], *Brachymystax lenok* juvenile [26], and *Apostichopus japonicus* juvenile [27]. Dietary lipid levels significantly altered whole-body ash content in bullfrog tadpoles. Gradually, the fore and hind extremities became visible as the structure transitioned from aquatic to terrestrial life. Therefore, the ash content of the whole body of bullfrog tadpoles increased [15].

The increase of intestinal digestive enzyme activities enhances the decomposition, digestion, absorption, and consumption of food by degrading macromolecules that are consumed by tadpoles [28]. Currently, there are more research results related to different and specific stages [28–31]. However, there is a paucity of research on the role of digestive enzymes in the different diets of tadpoles [32]. In the current study, the activities of digestive enzymes were not consistent across various diets. Gradually, the lipid levels in the diet led to an increase in LPS activity in the intestines of tadpoles. The elevated lipid levels in the diets and the need to synthesize additional LPS to metabolize the dietary lipid demonstrate a substrate dependence of intestinal LPS. This trend is in line with several earlier studies, such as *Scophthalmus maximus* L. [33], *A. japonicus* juvenile [27], *Babylonia areolate* [34], and *Clupea harengus* [35]. Various pancreatic enzymes have been reported to be essential for tadpole development and include TPS and AMS in *Xenopus laevis* [28]. The dietary lipid level of 10.90% was found to have an impact on increasing intestinal TPS activity in tadpoles, which indicated that an appropriate dietary lipid level might improve protein consumption. In this study, the intestinal AMS activity of tadpoles fed diets with 10.90%–15.00% lipid was substantially higher in the present study than that of tadpoles fed diets containing 4.46%–9.10% lipid. This difference may be attributed to changes in nutrient uptake by the tadpole digestive system [36, 37].

Blood biochemical indicators reflect the physiological and metabolic status of tadpoles and are closely associated with their nutritional status. The main form of lipids transported in the blood is TGs, whereas T-CHO plays an important role in cell membrane production, bile acid synthesis, vitamin D, and steroid hormone biogenesis [38, 39]. In this study, an active endogenous fat trafficking system, which is responsive to high-fat feeds, was indicated by the sustained increase in the serum T-CHO content of tadpoles. Further, significant indications of lipolysis and synthetic and hepatic fat metabolism were observed in the serum of tadpoles, including HDL-C and LDL-C. HDL-C plays a role in facilitating cholesterol transport and clearance in the body, and cholesterol is often transported to the liver for catabolism [40]. Here, the level of HDL-C increased from 4.46% to 12.34% as dietary lipid levels increased and then decreased. Based on these results, bullfrog tadpoles' potential to transfer cholesterol from extrahepatic tissues to the liver for catabolism is reduced by a high-fat diet.

The liver fulfills numerous functions, and lipid homeostasis in the liver is a complex process, including lipid uptake, transport, and metabolism [41]. To observe the deposition of fat in the liver of tadpoles, the liver was stained with Oil Red O. Fat deposition in the liver of tadpoles was observed to increase in correlation with the level of dietary fat. Further, the lipid synthesis and catabolism-related enzyme activities were assessed to obtain a comprehensive understanding of the role of dietary lipids in the liver in the regulation of lipid metabolism. The ACC is the speed-limiting enzyme in the synthesis of fatty acids [42]. The present results showed that ACC activity in the liver was reduced in high-lipid diet-fed tadpoles to inhibit fatty acid synthesis. Similar results were also found in *Ctenopharyngodon idellus* juvenile [2] and *Micropterus salmoides* [43]. Furthermore, LPL, a key enzyme in lipoprotein metabolism, plays an important role in lipolysis and has a direct role in regulating lipid metabolism and deposition in the body [44]. CPT-I is considered an essential regulatory enhancer in mitochondrial β-oxidation due to its ability to catalyze the transformation of fatty acyl-CoAs to fatty acyl-carnitine molecules, which allows it to access the mitochondrial substrate [45, 46]. In this study, the liver's LPL and CPT-I activities increased as the dietary lipid level increased from 4.46% to 12.34% and then decreased. These results illustrated lower dietary lipid levels led to inhibition of lipolysis in tadpoles. Therefore, disorders of fat metabolism may result from the consumption of either an excessive or insufficient amount of lipids. The trend was consistent with several earlier studies, such as *Oreochromis niloticus* [47], *Pelteobagrus vachelli* larvae [48], and *S. maximus* L. juvenile [49]. Therefore, the appropriate lipid levels could promote tadpole growth and enhance fat consumption and lipid metabolism.

Moreover, the livers are essential metabolic organs in aquatic animals, and their antioxidant function is closely associated with their overall health [50]. The potential of both antioxidant enzymes and nonenzymatic systems to adjust for external stimuli, as well as the state of radical formation in the system, are represented in T-AOC. Further, SOD promotes the O_2_^2−^ disproportionation, leading to the formation of H_2_O_2_ and molecular oxygen [51].The study found that the liver T-AOC potential increased when the dietary lipid levels were higher from 4.46% to 9.10% and then decreased. The same pattern was observed for the levels of SOD and CAT in the liver. These results suggested that tadpoles were susceptible to oxidative stress when their dietary lipid intake exceeded their body's requirements, resulting in a decrease in their antioxidant potential. This also clarified the progressive reduction of survival rate as the dietary lipid level increased. Comparable results have been found in the juvenile *Larimichthys crocea* [52], *Oncorhynchus mykiss* [53], and *S. maximus* L. [49]. These studies suggest that appropriate levels of dietary lipids may improve antioxidant function and scavenge reactive oxygen radicals, thereby reducing oxidative damage.

The anamorphic life cycle of tadpoles is more complex than that of other vertebrates, necessitating their metamorphosis [11, 54, 55]. Further, the development of growth and metamorphosis in vertebrates is particularly associated with thyroid hormones (THs) [56, 57]. THs bind to receptors on the thyroid gland, which then stimulate the synthesis and release of THs. T4 is one of the primary components of THs [57]. Particularly, T4 stimulates and induces the synthesis of a specific enzyme-activating protein to directly control amphibian metamorphosis [58]. Generally, the subarticular tumor forms a protrusion, and the cloacal orifice can be observed on its ventral side connected by a skin fold at growth stage 40. At growth stage 41, the tadpoles undergo critical developmental stages, including the loss of cloacal folds and the hyaline transition of the forelimb skin, before entering metamorphosis [15]. The present study determined that the tadpole serum contained higher T4 levels at 12.34% lipid content. The growth rate of bullfrog tadpoles during the 40th and 41st growth stages showed an increase followed by a decrease as the dietary lipid level increased from 4.46% to 12.34%. These findings illustrated that the normal nutrient and metabolism parameters were not met by individuals who consumed insufficient lipid diets and had low levels of T4. Another study also discovered that the metamorphosis of tadpoles occurred at a relatively slow rate when the feed nutrition was deprived [11]. Therefore, T4 is vitally essential in conditioning the morphology in the bullfrog tadpoles. The levels of lipids in diets can have an impact on the secretion of growth-regulating endocrine hormones by bullfrog tadpoles, which in turn affects tadpole metamorphosis.

In conclusion, based on the growth rate at stage 41 and the WG, the optimal dietary lipid level for bullfrog tadpoles was calculated to be 10.72%–11.08% of the diet. Further, bullfrog tadpoles are adversely affected by both deficits and excesses of lipids in their diets, which have a detrimental impact on their growth, antioxidant potential, and metamorphic processes.

## Figures and Tables

**Table 1:** Formulation and proximate composition of the experimental diets (% dry matter).

Ingredients (%)	Lipid levels (%)					
	4.46	6.95	9.10	10.90	12.34	15.00
Fish meal^a^	31.00	31.00	31.00	31.00	31.00	31.00
Soy protein concentrate	24.40	24.40	24.40	24.40	24.40	24.40
Wheat flour	27.82	27.82	27.82	27.82	27.82	27.82
Microcrystalline cellulose	10.00	8.00	6.00	4.00	2.00	0.00
Fish soluble	3.00	3.00	3.00	3.00	3.00	3.00
Soybean oil	0.00	2.00	4.00	6.00	8.00	10.00
Lecithin	0.80	0.80	0.80	0.80	0.80	0.80
Calcium dihydrogen phosphate	0.58	0.58	0.58	0.58	0.58	0.58
Calcium carbonate	1.00	1.00	1.00	1.00	1.00	1.00
Choline chloride	0.50	0.50	0.50	0.50	0.50	0.50
Mineral premix^b^	0.50	0.50	0.50	0.50	0.50	0.50
Vitamin premix^b^	0.20	0.20	0.20	0.20	0.20	0.20
L-Ascorbic acid polyphosphate	0.10	0.10	0.10	0.10	0.10	0.10
Yttrium(III) oxide	0.10	0.10	0.10	0.10	0.10	0.10
Proximate composition (%)						
Crude protein	45.30	45.30	45.50	45.60	45.60	45.60
Crude lipid	4.46	6.95	9.10	10.90	12.34	15.00
Crude ash	8.99	8.99	9.00	9.42	9.14	9.32
Total phosphorus	1.31	1.32	1.33	1.31	1.33	1.33
Calcium	1.56	1.57	1.59	1.57	1.58	1.57
Gross energy (MJ/Kg)	16.11	16.51	16.87	17.14	17.66	17.94

^a^Fish meal, Xiamen Jiakang Feeds Group Corp., Ltd., imported from Peru.

^b^Mineral premix and vitamin premix were prepared following our recent study [14].

**Table 2:** Growth performance and feed utilization of bullfrog tadpoles fed diets differing in protein levels.

Index	Lipid levels (%)						Pooled SEM	ANOVA	Linear		Quadratic	
	4.46	6.95	9.10	10.90	12.34	15.00		*p*-value	*p*-value	*R* ^2^	*p*-value	*R* ^2^
FBW (g)	3.74	5.28	6.38	6.45	6.73	5.47	0.17	<0.001	0.008	0.365	<0.001	0.899
WG (%, ×10^4^)	5.26	7.43	8.98	9.07	9.47	7.69	0.88	<0.001	0.008	0.365	<0.001	0.899
SGR (% day^−1^)	8.36	8.81	9.07	9.08	9.14	8.86	0.04	<0.001	0.006	0.391	<0.001	0.911
FCR	1.31	1.24	1.14	1.18	1.20	1.24	0.03	0.043	0.233	0.088	0.006	0.495
FI (%/day)	3.39	3.24	2.96	3.02	3.10	3.20	0.07	0.044	0.165	0.117	0.005	0.513
PER (%)	1.72	1.78	1.96	1.98	1.89	1.79	0.07	0.017	0.285	0.071	0.002	0.564
PDR (%)	11.74	12.08	13.94	18.25	17.54	13.73	0.04	<0.001	0.017	0.307	0.002	0.569
LDR (%)	28.65	30.77	33.90	36.28	31.09	17.04	0.71	<0.001	0.077	0.182	<0.001	0.843
Survival (%)	97.33	98.00	97.33	93.33	92.67	90.67	0.67	<0.001	<0.001	0.754	<0.001	0793

*Note:* Value was mean ± SEM (*n* = 3 for treatment). FBW (g) = final body weight. WG (%) = (final body weight − initial body weight)/initial body weight × 100. SGR (% day^−1^) = (ln final body weight − ln initial body weight)/feeding days × 100. FCR = dry feed intake/wet weight gain. FI (%/day) = 100 × dry feed intake/(final body weight/2 + initial body weight/2)/feeding days. PER (%) = 100 × wet weight gain/total protein intake. PDR (%) = 100 × (final body protein content − initial body protein content)/total protein intake. LDR (%) = 100 × (final body lipid content − initial body lipid content)/total lipid intake. Survival (%) = 100 × final number/initial number of bullfrog tadpoles.

Abbreviations: ANOVA, one-way analysis of variance; FBW, final body weight; FCR, feed conversion ratio; FI, feed intake; LDR, lipid deposition rate; PDR, protein deposition rate; PER, protein efficiency ratio; SEM, standard errors of the means; SGR, specific growth rate; WG, weight gain.

**Table 3:** Body composition and body index of bullfrog tadpoles fed increasing dietary protein levels.

Index	Lipid levels (%)						Pooled SEM	ANOVA	Linear		Quadratic	
	4.46	6.95	9.10	10.90	12.34	15.00		*p*-value	*p*-value	*R* ^2^	*p*-value	*R* ^2^
Whole body (fresh weight, %)												
Moisture	88.23	87.90	86.44	81.05	83.10	87.58	0.27	<0.001	0.090	0.169	0.002	0.554
Protein	6.81	6.77	7.11	9.25	9.29	7.69	0.30	<0.001	0.010	0.344	0.010	0.459
Lipid	1.63	2.59	3.42	4.47	4.46	3.07	0.18	<0.001	0.004	0.413	<0.001	0.823
Ash	1.04	1.09	1.16	1.61	1.54	1.08	0.06	0.001	0.118	0.146	0.014	0.433
Energy (MJ/Kg)	2.28	2.41	2.75	3.88	3.48	2.58	0.06	<0.001	0.043	0.233	0.001	0.604
Body index												
HSI^a^ (%)	3.05	3.15	3.30	3.54	3.50	3.17	0.06	0.003	0.068	0.193	0.002	0.570
AFP^b^ (%)	1.20	1.72	1.94	2.25	2.46	2.16	0.07	<0.001	<0.001	0.697	<0.001	0.883
CF^c^ (g/cm^3^)	0.73	0.74	0.78	0.80	0.82	0.89	0.02	<0.001	<0.001	0.756	<0.001	0.789

*Note:* Value was mean ± SEM (*n* = 3 for treatment).

Abbreviations: AFP, abdominal fat percentage; ANOVA, one-way analysis of variance; CF, condition factor; HSI, hepatosomatic index; SEM, standard errors of the means.

^a^HSI (%) = 100 × liver weight/final body weight.

^b^AFP (%) = 100 × abdominal fat weight/final body weight.

^c^CF (g/cm^3^) = 100 × final body weight/the body length^3^.

**Table 4:** Effects of dietary different lipid levels on digestive enzyme activity and apparent digestibility of bullfrog tadpoles.

Index	Lipid levels (%)						Pooled SEM	ANOVA	Linear		Quadratic	
	4.46	6.95	9.10	10.90	12.34	15.00		*p*-value	*p*-value	*R* ^2^	*p*-value	*R* ^2^
Digestive enzyme activity												
TPS (U/mgprot)	107.45	115.82	121.36	140.96	132.51	129.12	6.47	0.048	0.010	0.351	0.008	0.473
LPS (U/gprot)	1.75	1.93	2.24	2.23	2.52	2.34	0.13	0.025	0.001	0.498	0.002	0.563
AMS (U/mgprot)	0.23	0.25	0.25	0.48	0.43	0.44	0.03	0.001	<0.001	0.574	0.001	0.584
Nutrient digestibility												
Dry matter (%)	71.21	71.62	74.02	75.71	77.09	70.68	0.76	<0.001	0.255	0.080	0.007	0.488
Protein (%)	85.75	83.67	83.56	82.83	82.79	68.11	1.25	<0.001	0.001	0.508	<0.001	0.707
Lipid (%)	66.07	79.48	83.86	85.96	82.68	78.87	1.38	<0.001	0.025	0.277	<0.001	0.826
Energy (%)	72.61	73.41	75.53	75.91	78.69	71.54	0.66	<0.001	0.415	0.042	0.012	0.448

*Note:* Apparent digestibility coefficients (ADCs, %) = 100 × (1−F_0_/D_0_ × (D_Y_/F_Y_)). D_Y_ and F_Y_ are the concentration of yttrium in diet and feces, respectively, and D_0_ and F_0_ are the quantities of compositions in diets and feces, respectively. Value was mean ± SEM (*n* = 3 for treatment).

Abbreviations: AMS, amylase capacity; ANOVA, one-way analysis of variance; LPS, lipase; SEM, standard errors of the means; TPS, trypsin.

**Table 5:** Effects of dietary different lipid levels on the serum biochemical indexes of bullfrog tadpoles.

Index	Lipid levels (%)						Pooled SEM	ANOVA	Linear		Quadratic	
	4.46	6.95	9.10	10.90	12.34	15.00		*p*-value	*p*-value	*R* ^2^	*p*-value	*R* ^2^
T-CHO (mmol/L)	2.17	2.39	2.68	2.82	3.11	2.89	0.23	0.127	0.005	0.397	0.012	0.443
TG (mmol/L)	1.03	1.00	1.29	1.30	1.17	1.02	0.09	0.178	0.648	0.013	0.070	0.298
HDL-C (mmol/L)	0.41	0.44	0.71	0.78	0.96	0.67	0.06	0.002	0.003	0.431	0.001	0.586
LDL-C (mmol/L)	2.31	2.04	2.11	2.01	1.97	1.74	0.18	0.574	0.06	0.204	0.178	0.206

*Note:* Value was mean ± SEM (*n* = 3 for treatment).

Abbreviations: ANOVA, one-way analysis of variance; HDL-C, high-density lipoprotein cholesterol; LDL-C, low-density lipoprotein cholesterol; SEM, standard errors of the means; T-CHO, total cholesterol; TG, triglyceride.

**Table 6:** Effects of dietary different lipid levels on the liver biochemical indexes of bullfrog tadpoles.

Index	Lipid levels (%)						Pooled SEM	ANOVA	Linear		Quadratic	
	4.46	6.95	9.10	10.90	12.34	15.00		*p*-value	*p*-value	*R* ^2^	*p*-value	*R* ^2^
Lipid metabolism												
ACC (ng/gprot)	70.13	62.08	53.96	48.40	49.78	50.37	2.61	0.002	<0.001	0.591	<0.001	0.754
LPL (U/mgprot)	0.29	0.44	0.55	0.67	0.75	0.65	0.05	0.001	<0.001	0.641	<0.001	0.768
CPT-I (ng/gprot)	21.43	29.15	27.17	33.54	36.84	34.84	1.99	0.003	<0.001	0.606	<0.001	0.647
Antioxidant index												
T-AOC (mmol/gprot)	0.46	0.61	0.60	0.57	0.53	0.40	0.02	<0.001	0.170	0.114	<0.001	0.780
SOD (U/mgprot)	33.86	36.81	50.70	60.21	54.02	40.71	4.21	0.007	0.093	0.166	0.002	0.564
CAT (U/mgprot)	26.76	36.54	38.36	41.91	39.18	37.46	2.17	0.021	0.024	0.281	0.001	0.599
MDA (nmol/mgprot)	6.32	4.38	4.63	4.13	4.71	5.45	0.73	0.450	0.579	0.020	0.112	0.253

*Note:* Value was mean ± SEM (*n* = 3 for treatment).

Abbreviations: ACC, acetyl-CoA carboxylase; ANOVA, one-way analysis of variance; CAT, catalase; CPT-I, carnitine palmitoyltransferase I; LPL, lipoprotein lipase; MDA, malondialdehyde; SEM, standard errors of the means; SOD, superoxide dismutase; T-AOC, total antioxidant capacity.

## Data Availability

The data that support the findings of this study are available from the corresponding author upon reasonable request.
